# Artificial Intelligence Driving Materials Discovery? Perspective on the Article: Scaling Deep Learning for Materials Discovery

**DOI:** 10.1021/acs.chemmater.4c00643

**Published:** 2024-04-08

**Authors:** Anthony K. Cheetham, Ram Seshadri

**Affiliations:** † Materials Department and Materials Research Laboratory, University of California, Santa Barbara, California 93106, United States; ‡ Department of Materials Science and Engineering, National University of Singapore, Singapore 117575, Singapore

## Abstract

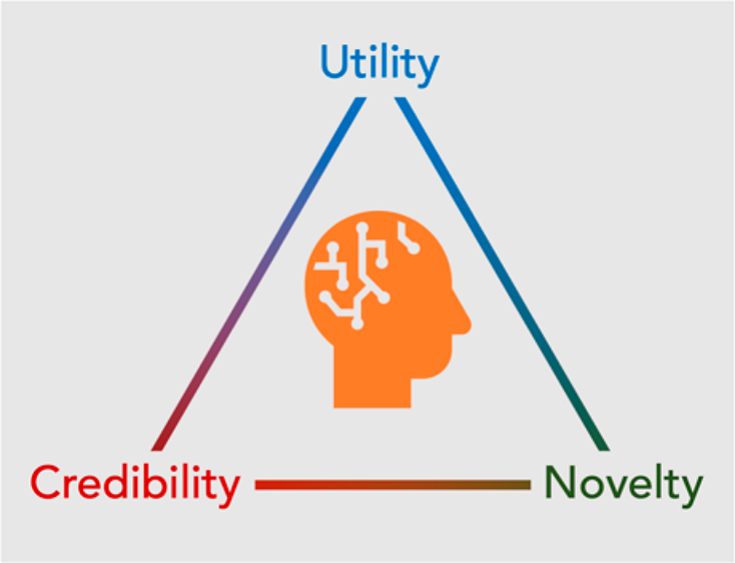

The discovery of new crystalline inorganic compounds—novel compositions of matter within known structure types, or even compounds with completely new crystal structures—constitutes an important goal of solid-state and materials chemistry. Some fractions of new compounds can eventually lead to new structural and functional materials that enhance the efficiency of existing technologies or even enable completely new technologies. Materials researchers eagerly welcome new approaches to the discovery of new compounds, especially those that offer the promise of accelerated success. The recent report from a group of scientists at Google who employ a combination of existing data sets, high-throughput density functional theory calculations of structural stability, and the tools of artificial intelligence and machine learning (AI/ML) to propose new compounds is an exciting advance. We examine the claims of this work here, unfortunately finding scant evidence for compounds that fulfill the trifecta of novelty, credibility, and utility. While the methods adopted in this work appear to hold promise, there is clearly a great need to incorporate domain expertise in materials synthesis and crystallography.

## Introduction

In an article in *Nature* published in November 2023, Merchant et al.^1^ describe the application of artificial intelligence and machine learning (AI/ML) techniques such as deep learning of experimental databases and computational data to the discovery of new inorganic materials, including classical inorganic compounds such as oxides and halides, as well as other main group compounds and intermetallics. Their approach claims to “*enable the discovery of 2.2 million structures below the current convex hull, many of which escaped previous human chemical intuition...representing an order-of-magnitude expansion in stable materials known to humanity*”. Almost 400,000 of the structures are deemed to be stable and have been listed in a Stable Structure database,^2^ while further structural details of more than 2000 of these new compounds have been placed in the GNoME Explorer archive^3^ within the Materials Project.^4^ The bold claims in the *Nature* paper warrant scrutiny by the materials chemistry community, which has hitherto undertaken the discovery of new materials through the (perhaps) more pedestrian approach of synthesizing new compounds planned around how they relate to what is already known, with experience and empirical knowledge (which is sometimes arguably mislabeled as chemical intuition) serving as guides. In this perspective, we scrutinize the claims by Merchant et al. by looking in detail at a small, randomized subset of the new materials in both the GNoME Explorer archive and the Stable Structure database to evaluate their novelty. We do this from the perspective of experimental materials chemists with decades of experience in discovery research who welcome new approaches to materials discovery.

We have recently contributed a perspective on the relationship between chemical synthesis and materials discovery wherein we have analyzed the different laboratory approaches that have led to major breakthroughs in the materials area, such as the discovery of high temperature superconductivity and lithium ion batteries.^5^ One of the lessons from that study was that most breakthroughs are not achieved by design or serendipity, but by exploring opportunities in the extensive repository of compounds that are already known. In this respect, we applaud the news that this repository has apparently become ten times larger since this should clearly increase the probability of making important materials breakthroughs in the future. The following sections summarize our views on this potentially important development.

Before progressing further, we point out that the predictions in the contribution of Merchant et al.^1^ are solely of crystalline inorganic compounds and should be described as such, rather than using the more generic label “material”. There are many communities who would justifiably be upset by the description “*representing an order-of-magnitude expansion in stable materials*”: polymers, glasses, metal–organic frameworks, heterostructures, and composites are only a few excluded materials classes that come to mind, and that are each infinite in their scope. Additionally, it is the usual practice in the field that chemical compounds become materials when they demonstrate some utility. We propose that impactful predictions of new materials should lie somewhere within the triangle of being credible, implying that the proposed structure and composition of matter should be experimentally realizable, novel in the sense of not being more than a trivial extension of known compounds, and display some evidence of utility so that they can truly be recognized as materials.

## General Comments about the GNoME Explorer Database

In this first section, we have looked through part of the GNoME Explorer database for any obvious signs of entries that may lack novelty. This has not been done comprehensively since this would be too time-consuming, but we examined the first 250 entries to get a sense of any underlying issues. Each of the 2047 entries in the GNoME Explorer compilation has been presented with chemical composition, space group, atomic coordinates, formation energy, and, where appropriate, tentative assignments of oxidation states. In the spirit of constructive criticism, we make the following observations concerning the compounds in this subset of the GNoME database.

(i) We believe that experimentalists, who presumably are an important target audience, would find it helpful if the results were presented in a more organized manner, rather than as a seemingly random walk through the periodic table. For example, it would be useful to list all the oxides together, as well as the fluorides, chlorides, bromides, etc. This could no doubt be done by the user with the help of the search function in the database, but it would be helpful if it was done in the parent listing, given the enormous number of entries.
(ii) The compositions are often not presented in a manner that an experimental materials chemist would find appropriate or helpful, nor are the usual rules of chemical nomenclature followed, notably, anions after cations, alkali and alkaline earth metals before transition metals, etc. For example, Ac(ErB_8_)_3_ (mp-3169969) could be more usefully listed as AcEr_3_B_24_ since there is no special relationship between the Er and the B. It is then easy to see that this is a hexaboride of Ac and Er: AcEr_3_(B_6_)_4_. Such hexaborides are well-known and are found for a wide range of elements, such as the alkaline earths and rare earths. LaB_6_ is a typical example and adopts the cubic space group, *Pm*3̅*m*, with *a* ≈ 4.1 Å.^6^ AcEr_3_(B_6_)_4_ is predicted to be tetragonal with *a* ≈ 4.1 Å and *c* ≈ 16.5 Å, revealing that a proposed ordering of Ac^3+^ and Er^3+^ (which may or may not be experimentally viable) leads to a 4× superstructure along *c* and lowers the symmetry of the parent LaB_6_ structure from cubic to tetragonal. There are other examples of apparently novel and complex borides that are based upon the LaB_6_ structure, e.g., K_2_SmLuB_24_ (mp-3170680), but the unconventional presentation of their compositions makes it difficult to search for them automatically. Perhaps this could be addressed in the next iteration of the database.
(iii) Oxidation states are often inconsistent with known materials, or structural features are obscure or entirely unprecedented in known materials. For example, TbSmF_30_ (mp-3170019) defies all the usual rules of valence and contains an improbable ordering of Tb^3+^ and Sm^3+^ (see below). Further inspection of the structure reveals that it contains large numbers of F_2_ molecules, some isolated and others acting as linkers between *Ln*F_9_ polyhedra (where *Ln* is the lanthanide cation). Neither of these features is found in the literature on known fluoride compounds, and their existence at ambient pressure seems highly improbable through a structural chemist’s lens. There are other examples in the database of compositions with a large excess of anions that potentially arise from the difficulty of dealing with the chemical potential of a gas-phase component (in this case F_2_) in electronic structure calculations of formation energies and stability.^7^
(iv) Several of the proposed new structures have obvious analogues, which somewhat undermines the impact of their prediction. K_3_Nd(AsO_4_)_2_ (mp-3196061) is a good example. It is predicted to be monoclinic, in space group *P*2_1_, with *a* = 9.8 Å, *b* = 5.8 Å, *c* = 7.6 Å, and β = 91.87°. Since arsenates are often isostructural with phosphates, it is possible to recognize that the analogous phosphate, K_3_Nd(PO_4_)_2_, is known with a monoclinic *P*2_1_/*m* structure (*a* = 9.5 Å, *b* = 5.6 Å, *c* = 7.4 Å, β = 90.95°).^8^ These structures are virtually identical, aside from the lower symmetry in the GNoME entry, a recurring theme that we shall return to below.
(v) We would now like to focus on a recurrent issue that is found throughout the GNoME database, which is that many of the entries are based upon the ordering of metal ions that are unlikely to be ordered in the real world. For example, it is well-known that ordering of lanthanide atoms and ions is highly improbable at finite temperatures. However, density functional theory (DFT)-based electronic structure calculations at 0 K will find a way to order them because they do not take entropy into account. Entropic effects, however, typically favor disordered arrangements at the synthesis temperatures used for most inorganic materials. For example, in TbSmSeO_2_ (mp-3169970), the Tb^3+^ and Sm^3+^ cations are predicted to be on separate crystallographic sites, even though their charges are the same and their 6-coordinate ionic radii are very similar (0.92 and 0.96 Å, respectively). Because of the ordering, the compound is proposed to be non-centrosymmetric in space group *P*3*m*1 with *a* ≈ 3.91 Å and *c* ≈ 6.92 Å. Unfortunately, this improbable rare-earth ordering obscures the fact that the disordered structure of (Tb/Sm)SeO_2_ would be isomorphous with the known *centrosymmetric P*3̅*m*1 structure of La_2_SeO_2_, which has *a* ≈ 4.09 Å and *a* ≈ 7.16 Å.^9^ As in the case of Ac(ErB_8_)_3_, discussed above, the artificial ordering of the cations lowers the symmetry from a centrosymmetric to a non-centrosymmetric structure, albeit within the same crystal system. We regard these as predictions that are highly unlikely to be confirmed because of the virtual certainty that the cations will be disordered.
(vi) The above comments about cation ordering in inorganic compounds also apply to a very large number of the intermetallic phases, leading to many other examples of unlikely predictions. For example, DyHo_2_Rh_9_ (mp-3170148) is predicted to adopt space group *P*6_3_/*mmc* with *a* = 5.26 Å and *c* = 17.58 Å. This compound clearly has the PuNi_3_ structure, which is found in a large number of phases such as CeNi_3_, *P*6_3_/*mmc* with *a* = 4.98 Å and *c* = 16.94 Å.^10^ Ordered variants of this structure are also known, e.g., YRh_2_Si (*P*6_3_/*mmc* with *a* = 5.49 Å and *c* = 15.03 Å),^11^ though it seems highly improbable that Dy and Ho would order in the proposed DyHo_2_Rh_9_ GNoME structure. TbHo_2_Tm_9_Co_4_ (mp-3170547) is an even more striking example among the intermetallics. This proposed alloy between three smaller rare earths and cobalt is predicted to be monoclinic and non-centrosymmetric, space group *Pm*, with *a* = 6.14 Å, *b* = 9.23 Å, *c* = 6.89 Å, and β = 90.1°. If we rewrite the formula as Ln_12_Co_4_, then we can see that it represents an unlikely ordering of the small lanthanides in a compound of composition Ln_3_Co. These phases are known for most of the rare earths and yttrium, and they adopt the Fe_3_C structure. For example, Ho_3_Co adopts a centrosymmetric orthorhombic structure, space group *Pnma*, with *a* = 6.92 Å, *b* = 9.29 Å, and *c* = 6.21 Å;^12^ note that the *a* and *c* axes are inverted and that the lowering of symmetry in the AI prediction is again due to the artificial ordering of the small rare-earth metals.
  There appear to be countless similar examples in the GNoME database. For example, Tb_16_Ho(ErIr_4_)_3_ (mp-3170203) could be more usefully written as Tb_16_HoEr_3_Ir_12_. It is predicted to be tetragonal, *P*4̅ (non-centrosymmetric), with *a* = 10.92 Å and *c* = 6.38 Å. If we assume that very similar rare earths would be disordered, as discussed above, it can then be written as Ln_20_Ir_12_ or Ln_5_Ir_3_. This is a known structure type and has been reported for virtually all rare earths with the tetragonal Pu_5_Rh_3_ structure. For the specific case of Tb_5_Ir_3_,^13^ the space group is centrosymmetric *P*4/*ncc* with *a* = 10.905 Å and *c* = 6.299 Å. Note again that the prediction places the different lanthanides on distinct sites and therefore lowers the symmetry. We will discuss the reasons for the frequent lowering of symmetry that is found in many of the Stable Structure database entries in a later section.
(vii) Another common claim among the database entries is the prediction of new compounds based upon radioactive elements that do not occur in usable quantities in nature. In the case of the proposed actinium (Ac^3+^) compounds, of which there are 27 (and approximately 6754 in the larger Stable Structure database), it should be noted that all isotopes of actinium are intensely radioactive with half-lives ranging from days to a few years.^14^ The natural abundance of Ac in, for example, uranium ores is so low that a ton of uranium yields less than a milligram of ^227^Ac (half-life 21.77 years). Milligram quantities of ^227^Ac can be made artificially by neutron irradiation of ^226^Ra, and this isotope has been studied for cancer treatments. However, ionic actinium compounds, which always contain Ac^3+^, have no industrial applications. Furthermore, the few that are known are isomorphous with their stable lanthanum analogues (e.g., AcPO_4_ and LaPO_4_). The “discoveries” of new actinium compounds, such as AcPO_3_ (mp-3170055), are therefore impractical and not very novel. In fact, there are 18,138 compounds of such radioactive elements in the large Stable Structure database, including those of Pm, Ac, and Pa. We question whether these can be regarded as potential new materials. There are a further 23,529 entries for compounds containing the highly radioactive elements Tc, Np, and Pu.

## Space Group Analysis

Considering the frequent observations of unlikely low symmetry structures among the predictions, we have examined the space group statistics of the 384,870 entries in the Stable Structure database. We have found that the space groups of the predicted compounds (Table 1) have a distribution that is strikingly different from those in the Inorganic Crystal Structure Database (ICSD),^15^ which has been analyzed by Urusov and Nadezhina.^16^ For example, the top two space groups in the ICSD are centrosymmetric *Pnma* and *P*2_1_/*c*, accounting for approximately 16% of all structures, with ∼8% in each case. By contrast, *Pnma* is ranked 16th in Stable Structure database with 1.56% of the structures, while *P*2_1_/*c* is found in only 0.7% of the structures, which is an order of magnitude lower than in the ICSD. In fact, the top four space groups in the Stable Structure database are all non-centrosymmetric and account for ∼34% of all the structures. By contrast, in the ICSD there is only one non-centrosymmetric space group in the top 24 and it accounts for only 1% of all structures.

**Table 1:** Most Abundant Space Groups in the Stable Structure Database

Stable Structure Database (384,870 entries)						
Space group	Occurrence	Percentage		Space group	Occurrence	Percentage
*Pm*	49037	12.7		*R*3̅*m*	8834	2.30
*P*1	39382	10.2		*P*6̅	8563	2.22
*Amm*2	26467	6.88		*P*6̅2*m*	7652	1.99
*Cm*	19913	5.17		*Imm*2	7394	1.92
*C*2/*m*	12954	3.37		*P*3*m*1	6903	1.79
*R*3*m*	11241	2.92		*P*3̅*m*1	6379	1.66
*C*2	11201	2.91		*P*6̅*m*2	6038	1.57
*P*1̅	10463	2.72		*Pnma*	6005	1.56

The striking disparity between the space group distributions for the predicted phases and known ICSD structures is a matter of serious concern. We also note that this issue has been highlighted^17^ in a commentary on a different *Nature* article on robotic/AI-based materials discovery.^18^ The main reason for the disparity is due to the frequent prediction of structures with atoms ordered on distinct crystallographic sites that—in most cases—are likely to be disordered. In many cases, e.g., Dy_6_Y_2_Ho_11_Lu(Cd_3_Ru)_2_ (mp-3195118), they are suggestive of high-entropy alloys,^19^ where the whole point is to avoid atomic ordering or phase separation. Predictions of ordering usually lead to lower symmetry structures and in some cases to superstructures, as illustrated above. These general trends that are not found in the laboratory could arise because the DFT modeling is performed at effectively 0 K, and on small unit cells, ignoring the effects of configurational entropy, resulting in the predicted structures being fully ordered. In addition, many compounds undergo phase transitions to lower symmetry structures on cooling to low temperatures, although we have no evidence of this from the GNoME entries that we have examined.

## Randomly Selected Examples from the Stable Structure Database

Because our scrutiny of the GNoME database was selective to identify some obvious shortcomings of the methodology, we have also carried out a random examination of some of the entries in the Stable Structure database, selecting 10 compounds from among the 384,870 database entries. The results from this analysis are summarized in Table 2.

**Table 2:**
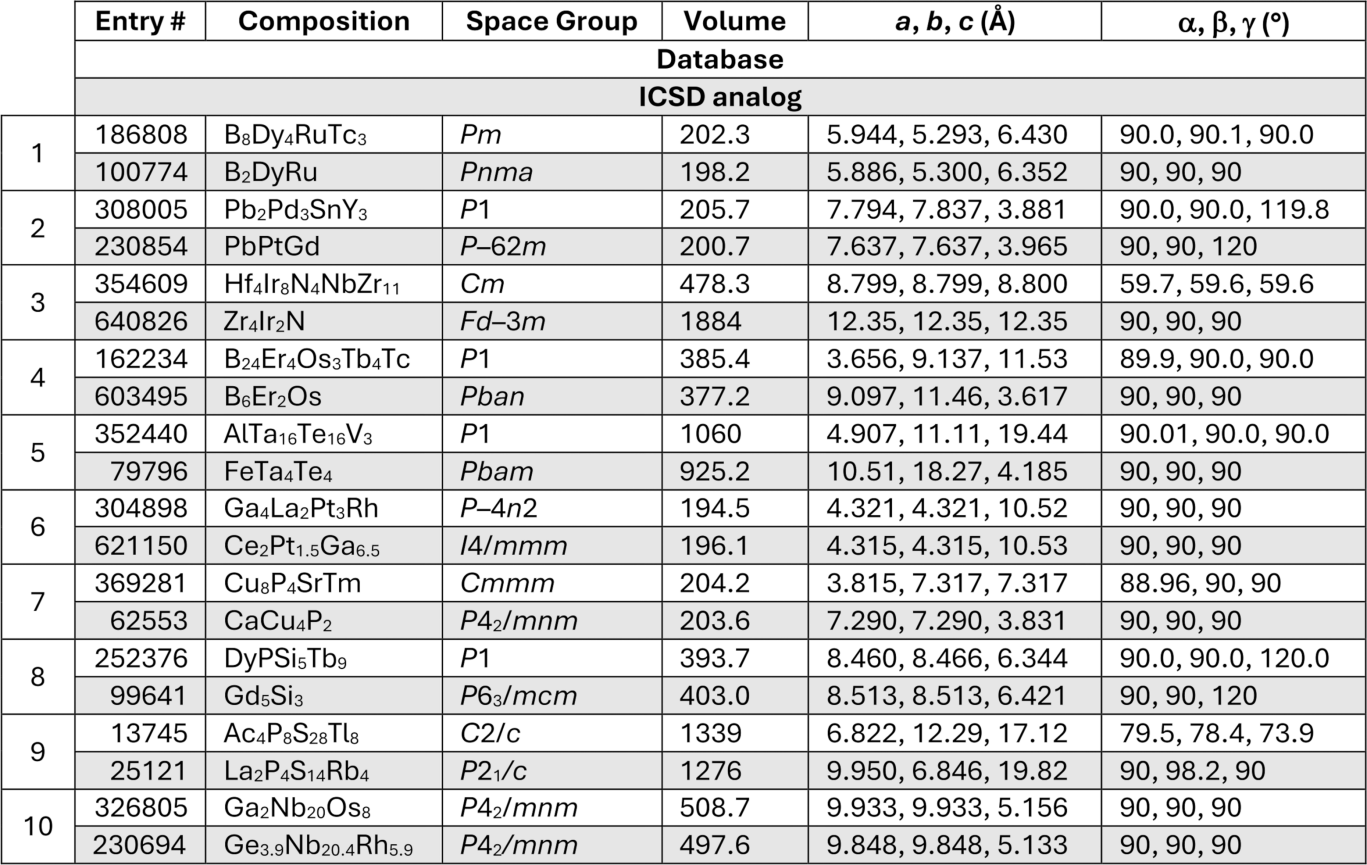
Comparison of 10 Randomly Selected Compounds from the Stable Structure Database with Appropriate ICSD Entries^a^

a The high incidence of pseudosymmetry in the Stable Structure database entries is notable.

**Figure 1:**
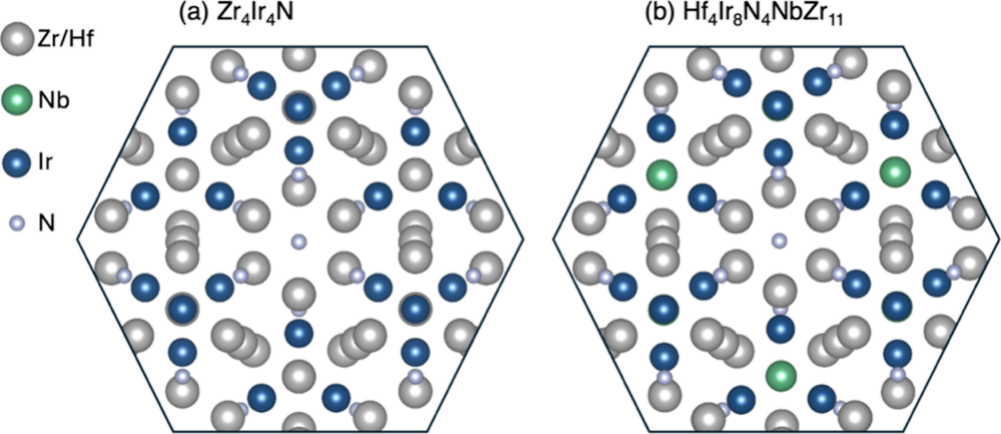
Views of the crystal structures of (a) known Zr_4_Ir_4_N (ICSD 640826) compared in a similar projection and identical scaling with (b) the proposed structure of Hf_4_Ir_8_N_4_NbZr_11_. The novelty of the structure and composition in (b) would arise only if Zr, Hf, and Nb were ordered on distinct crystallographic sites, which is unlikely.

The key conclusions from this analysis are as follows:

(i) We were able to identify the structure of every one of the 10 Stable Structure entries in the ICSD database, albeit usually with a space group of higher symmetry than that in the AI database. This is expected from the discussion above, with the lowered symmetry arising from artificial atomic orderings, and indeed, several of the Stable Structure entries involve unit cell parameters that are incompatible with the stated space groups, i.e., display pseudosymmetry.
(ii) In only one case was the space group the same. This enabled us to find an obvious analogue of Ga_2_Nb_20_Os_8_ in the ICSD by searching for structures in space group *P*4_2_/*mnm* with lattice parameters close to *a* = 9.9 Å and *c* = 5.1 Å, pointing us to Ge_3.9_Nb_20.4_Rh_5.9_, which is one of the well-known Frank–Kasper sigma (σ) phases.^20,21^ This is a common and versatile intermetallic structure type that is well-known to accommodate disorder on the five independent metal sites in the unit cell. It is indeed impressive that the predictive approaches can identify new compositions in this structure space.
(iii) In some cases, the analogy between the Stable Structure database and the ICSD entries is obvious. For example, Cu_8_P_4_SrTm was identified as having the same structure as CaCu_4_P_2_,^22^ noting that the lattice parameters were switched between the two entries and that the predicted compound was pseudo-tetragonal. It is questionable whether Sr and Tm would order on distinct crystallographic sites. If Tm were to substitute as the expected Tm^3+^, this would require electron-doping a Cu^1+^ compound, which is unlikely.
(iv) In other cases, the identification of the structure type takes a little more knowledge of the periodic table and crystal symmetry. For example, in Hf_4_Ir_8_N_4_NbZr_11_ our starting point was to recognize that Hf and Zr are two of the most similar elements in the periodic table and are almost certain to be disordered on the same sites (pointing us toward Zr_11_Ir_8_N_4_Nb). Furthermore, in a metal-rich compound, Nb and Zr/Hf are also likely to alloy on the same crystallographic site. A search in the ICSD for compounds containing Zr, Ir, and N then quickly led us to Zr_4_Ir_2_N,^23^ whose cubic unit cell is 4× larger than the cell of Hf_4_Ir_8_N_4_NbZr_11_ in the database. The final step is to recognize that the pseudo-rhombohedral cell from the Stable Structure database with α = 59.7° is also nearly pseudocubic, thus accounting for the 4× discrepancy in the cell volume. The two structures are compared in Figure 1.
(v) In the case of Ac_4_P_8_S_28_Tl_8_, the similarity to the structure of La_2_P_4_S_14_Rb_4_^24^ is not immediately apparent from the unit cells. However, we would expect Ac compounds would be isomorphous with their La analogues. Tl^1+^ and Rb^1+^ are also close in size and identical in their charge states. The similarity of the structures then becomes evident.

## Conclusions

Our analysis of the predictions by Merchant et al.^1^ has raised several important issues that need to be addressed for AI to have a significant impact on the discovery of new materials. These include the elimination of a large number of radioactive materials that are unlikely to have any utility in the materials world. This point particularly concerns the inclusion of compounds of Pm, Ac, and Pa (more than 18,000 in all), which are only available in minute quantities and in the rarest of circumstances. The challenges of dealing with disorder and behavior at finite temperatures are far more daunting. The computational tools to deal with these issues do exist, but they are computationally intensive and are not scalable in how Merchant et al.^1^ have approached their work. Much could be achieved, however, by embedding a knowledge of solid-state chemistry into their methodology. For example, the recognition that the 14 rare-earth elements and yttrium have very similar chemistries could be incorporated as well as the recognition that zirconium and hafnium, among other important metal pairs, have virtually identical chemistries.

There is also much room for improvement in the crystallographic aspects of the work. For example, ICSD has excellent search options that can utilize combinations of elemental composition, lattice parameters, and space groups. We employed these to identify the structures that are listed in Table 2. There are several crystallographic tools available to regularize structural information and enable the comparison of seemingly distinct structure types.^25^ Some of them would be useful in resolving issues of pseudosymmetry that is present in many of the entries.^26^ Efforts to relate structure types using their structures and geometries are also ongoing, and clearly need large-scale implementation.^27^

While the above analysis may seem to be critical, we do believe that many of our points could be adopted in the next version of this work. More scrutiny of the “new” materials needs to be performed prior to putting them into a database and claiming “...*an order-of-magnitude expansion in stable materials known to humanity*”. In fact, we have yet to find any strikingly novel compounds in the GNoME and Stable Structure listings, although we anticipate that there must be some among the 384,870 compositions. We also note that, while many of the new compositions are trivial adaptations of known materials, the computational approach delivers credible overall compositions, which gives us confidence that the underlying approach is sound. For example, in addition to the hexaborides, there are many examples in the GNoME list that are clearly diborides, which is another important stoichiometry in the boride area. What is now needed is greater effort to connect the predictions to what is already known in the literature to filter out the many candidates that are not truly novel. It is impractical to do this manually with a list of almost 400,000 new compositions.

It also must be recognized that a large fraction of the 384,870 compositions adopt structures that are already known and can be found in the ICSD database. This is not surprising to an experimental materials chemist, because it is quite rare to find an entirely new structure type in the inorganic world. This can be thought of as being a manifestation of Pauling’s Fifth Rule, the Rule of Parsimony.^28^ According to this rule, the number of geometrical units that Nature uses to assemble crystals is quite limited and relies heavily on a small number of recurrent polyhedral motifs such as tetrahedra or octahedra. While we are sure that there must be some new structure types predicted, our analysis suggests that there will not be many of them.

This brings us to our final point concerning the claim of “*an order-of-magnitude expansion in stable materials known to humanity*”. We would respectfully suggest that the work by Merchant et al.^1^ does not report any new materials but reports a list of proposed compounds. In our view, a compound can be called a material when it exhibits some functionality and, therefore, has potential utility. Since no functionality has been demonstrated for the 384,870 compositions in the Stable Structure database, they cannot yet be regarded as materials. The few examples of functionality mentioned in the article are associated with Li^+^-ion conductors. While the proposed materials are encouraging, their compositions leave much to be desired since they incorporate chemically soft anions. These anions are usually associated with narrow electrochemical stability windows, which renders materials that incorporate them somewhat pointless as Li^+^ solid electrolytes.^29^ This points to an interesting contraindication in materials design: soft anions such as Te^2–^ and I^–^ readily permit cation transport but do not possess the requisite electrochemical (redox) stability for use as solid electrolytes. Conversely, hard anions such as O^2–^ and F^–^ are redox-stable, but they bind cations strongly, and the resulting materials usually display limited ionic conductivity.

In closing, we hope the comments presented here will usefully serve the large community of materials scientists and engineers in their continued quest to develop the next generation of useful materials. While we are confident that the tools of Artificial Intelligence and Machine Learning have a bright future in the field of materials discovery, more work needs to be done before that promise is fulfilled.
